# Interface-Dominated Time-Dependent Behavior of Poled Poly(Vinylidene Fluoride–Trifluoroethylene)/Barium Titanate Composites

**DOI:** 10.3390/ma13010225

**Published:** 2020-01-04

**Authors:** Sara Dalle Vacche, Dragan Damjanovic, Véronique Michaud, Yves Leterrier

**Affiliations:** 1 Laboratory for Processing of Advanced Composites (LPAC), Ecole Polytechnique Fédérale de Lausanne, EPFL-STI-IMX-LPAC, Station 12, CH-1015 Lausanne, Switzerland; veronique.michaud@epfl.ch; 2 Group for Ferroelectrics and Functional Oxides, Ecole Polytechnique Fédérale de Lausanne, EPFL-SCI-STI-DD, Station 12, CH-1015 Lausanne, Switzerland; dragan.damjanovic@epfl.ch

**Keywords:** poly(vinylidene fluoride–trifluoroethylene), PVDF, barium titanate, ferroelectric composites, piezoelectric coefficient, interfacial interactions, silanes, coupling agents

## Abstract

Composites in which particles of ferroelectric ceramic phase are randomly dispersed in a polymeric matrix are of interest because of flexibility, conformability, and ease of processing. However, their piezoelectric properties are rather low, unless very high volume fractions of ceramics are used. This brings agglomeration and porosity issues due to the large mismatch between the surface energies of the ceramics and of the polymer. Particle surface modification is a common approach for better dispersion; however, it may bring other effects on the properties of the composites, which are usually concealed by the huge improvement in performance due to the more homogenous microstructure. In this work, we compared poly(vinylidene fluoride–trifluoroethylene)/barium titanate composites containing 15 vol.% and 60 vol.% of pristine ceramic particles or particles modified with an aminosilane or a fluorosilane. Similar morphology, with good particle dispersion and low porosity, was achieved for all composites, owing to an efficient dispersion method. The materials were poled with two different poling procedures, and the piezoelectric coefficient d_33_, the relative permittivity, and the poling degree of barium titanate were followed in time. We highlighted that, although similar d_33_ were obtained with all types of particles, the nature of the particles surface and the poling procedure were associated with different charge trapping and influenced the evolution of d_33_ with time.

## 1. Introduction

Ferroelectric materials, which possess piezoelectric and pyroelectric properties, may be used to convert thermal and mechanical energy into electric energy, and vice-versa. Particularly, their piezoelectric activity offers the opportunity of harvesting energy from, e.g., human motion, vibrations, and other forms of mechanical energy otherwise wasted in the environment. Other common applications include sensors and actuators. Common ferroelectric materials are perovskite ceramics such as lead zirconate titanate (PZT) and barium titanate, and, among polymers, polyvinylidene fluoride (PVDF) and its copolymers, and odd numbered nylons [1,2,3,4,5]. For a few decades, a steadily growing research effort is devoted to polymer matrix composites containing ferroelectric ceramic fillers, such as PZT or, due to environmental and health concerns, lead-free alternatives as barium titanate or potassium sodium niobate (KNN) [6,7]. PVDF-based polymers are considered the matrix of choice due to their high permittivity, favoring the poling process, and for their ferroelectric properties allowing for interesting poling possibilities [8]. In particular, 0–3 composites, in which the ceramic particles are randomly dispersed in the polymeric matrix, are an interesting option for large scale production and applications due to their flexibility, conformability, and ease of processing, although they have lower piezoelectric properties than ceramics.

To obtain 0–3 composites with interesting piezoelectric properties, high volume fractions of ceramic particles, which have high surface energy, are introduced in a polymeric matrix with much lower surface energy, with consequent problems such as particle agglomeration and porosity [9,10]. As a very common approach for achieving better dispersion, and hence lower porosity and better dielectric and mechanical properties, coupling agents, including, e.g., silanes or phosphonic acids [11,12,13,14], are used to increase the compatibility between the ceramic particles and the matrix, by decreasing the surface energy of the particles, and in some cases creating bonds with the matrix. Silanes and phosphonic acids are commercially available bearing a wide range of functional groups on their non-hydrolyzable organic chain, as e.g., alkyl, amino, or fluorinated groups. Several of them were found to enhance the dispersion of the ceramic particles in PVDF (co)polymer composites, resulting in improved properties compared to those of composites made with pristine particles [12,13,14,15,16,17,18].

However, also in view of upscaling of the production for industrial applications of polymer/ceramic composites, an effort is made to better understand if the particle surface modification is the best option for obtaining good particle dispersion, and which other consequences it may entrain for the functional properties of the composites. Indeed, the large morphology differences usually existing between composites with pristine and surface-modified fillers may hide some detrimental effects of the surface modification, which are mitigated by the large dispersion improvement. In order to obtain a better comparison, highly efficient dispersion techniques are used to obtain well-dispersed composites with pristine particles, and they are compared with composites with similar morphology, containing surface-modified particles [19,20]. Furthermore, although all the typically used organic surface modifiers generally bring a reduction of surface energy that reduces agglomeration, an important open question is how the functional groups contained in the surface modifiers affect the interfacial interactions and hence the properties of the composites. Contributing to the effort of clarifying these issues, we recently compared P(VDF–TrFE)/BaTiO_3_ composites with up to 60 vol.% of pristine particles and of particles modified with three silanes differing for the functional group at the end of the aliphatic chain (–CH_2_NH_2_, –CF_3_, –CH_3_). We showed that the presence of silanes on the surface of the particles generally resulted in a decrease of permittivity compared to pristine particles, which competes with the permittivity increase that may result from reduced porosity. Furthermore, we showed that the nature of the functional group on the silane chain strongly influenced the properties, as only the silane with an amino group was able to completely suppress the low-frequency dielectric losses and increase the thermomechanical stability, while fluorinated and alkyl silanes did not [21].

The piezoelectric response of 0–3 polymer/ceramic nanocomposites depends on several factors, including the volume fraction and aspect ratio of the particles, their distribution inside the polymer matrix, and the poling conditions. The stability in time of the piezoelectric coefficient is also an important factor to keep into account for industrial applications. The piezoelectric response of PVDF and barium titanate have been found to generally have a very moderate logarithmic decrease in time. On the other hand, very little research is available on the temporal evolution of the piezoelectric response in composites. Some works on this topic however point out that the interface between polymer and particles plays an important role in the stability of the piezoelectric response. Indeed, for 1–3 epoxy/PZT composites for which the ceramic pillars had a high surface to volume ratio, a higher decrease in time (about 20% in 500 days) of the piezoelectric coefficient was observed than for low surface to volume ratios [22].

In previous work, we observed, but could not elucidate, the difference in the value and temporal evolution of the piezoelectric coefficient of P(VDF–TrFE)/barium titanate composites when an aminosilane was used to improve the dispersion of the particles [23]. In this work, we focus on the effect of surface modification on the poling and the long-term piezoelectric response of P(VDF–TrFE)/barium titanate composites. We prepared composites containing particles in their pristine form or surface-modified with two silanes, differing only for the nature of the functional group at the end of their alkylic chain: we chose an aminosilane, that can bear a positive charge and create hydrogen bonds with the polymer, and a fluorosilane, that should minimize the polarity difference with the fluorinated polymer matrix. We particularly took care of obtaining good particle dispersion, low porosity, and similar structure and morphology for all composites to minimize effects due to these factors. We studied composites with two ceramic volume fractions (i.e., 15 vol.% and 60 vol.%), and for the high ceramic volume fraction composites, we used two different poling procedures. After poling, we followed the evolution of the piezoelectric coefficient d_33_, of the relative permittivity, and of the poling degree of BaTiO_3_ through XRD measurements, over a time of more than 100 days. We finally used thermally stimulated discharge measurements to check for the presence of accumulated charges and electret effects.

## 2. Materials and Methods 

### 2.1. Materials

**Figure 1:**
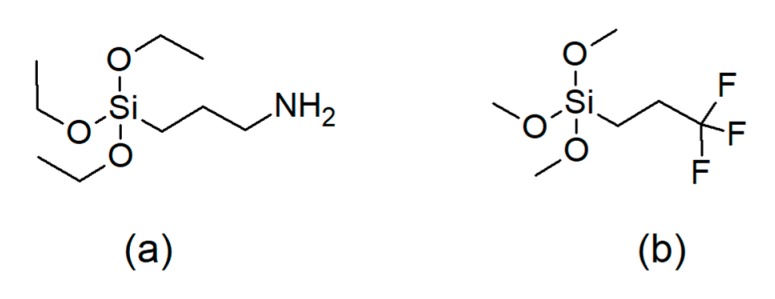
Structures of the silane coupling agents: (**a**) (3-aminopropyl)triethoxysilane and (**b**) (3,3,3-trifluoropropyl)trimethoxysilane.

The P(VDF–TrFE) (77/23 mol%) copolymer in powder form was provided by Solvay Solexis SpA (Bollate (MI), Italy). Barium titanate (BaTiO_3_), 99.95%, electronic grade, average particle size 0.2 μm, was purchased from Inframat Advanced Materials LLC (Manchester, NH, USA). The silane coupling agents used for the surface modification, both supplied by Sigma-Aldrich (St. Louis, MO, USA) were (3-aminopropyl)triethoxysilane, 99% and (3,3,3-trifluoropropyl)trimethoxysilane, 97%. The structures of the silanes are shown in Figure 1. Methyl ethyl ketone (MEK), 99+%, and ethanol, 99.5%, were purchased from Acros Organics (Geel, Belgium).

### 2.2. Methods

The barium titanate particles were surface-modified in an ethanol/water solution (95/5 *vol/vol*) for 2.5 h at 70 °C, rinsed, and dried at 110 °C for 30 min to promote silanol condensation. The details of the procedure are described in [21]. The pristine barium titanate particles will be indicated in what follows with BT, while the particles modified with the aminosilane or with the fluorosilane will be indicated with BTA and BTF, respectively. 

Composite films of P(VDF–TrFE), 40–50 μm thick, containing 15 vol.% and 60 vol.% of BT, BTA, and BTF particles were prepared by solvent casting: the particles were dispersed mechanically (Ultra-Turrax T25 digital disperser, IKA Werke GmbH & Co. KG, Staufen im Breisgau, Germany) and by ultrasounds (Sonifier 450 digital ultrasonic horn, Branson Ultrasonics Corporation, Danbury, CT, USA) in a 10 wt% solution of P(VDF–TrFE) in MEK at 60 °C. After degassing in vacuum, the mixtures were cast on glass and dried in a vacuum oven at 80 °C for 4 h. The films were annealed at 135 °C for 30 min to increase crystallinity. The details of the procedure are reported in [21]. The composites containing BT, BTA, and BTF will be indicated with pBT, pBTA, and pBTF.

A Merlin (Carl Zeiss A.G., Oberkochen, Germany) ultra-high resolution field emission scanning electron microscope (FESEM) was used to observe the particles and the cross-sections of the composites. The composite specimens were prepared with an Ilion II+ Model 697 precision ion polishing mill (Gatan Inc., Pleasanton, CA, USA). The milling conditions were 5 kV tension, temperature of −100 °C, 1° beam inclination, and polishing angle of 70°. The polished cross-sections and the particles spread on carbon tape were carbon-coated before FESEM observation to prevent charging with a 208carbon high vacuum carbon coater (Cressington Scientific Instruments Ltd., Watford, UK). The thickness of the coating was approximately 15 nm, assessed with an MTM-10 Thickness Monitor (Cressington Scientific Instruments Ltd., Watford, UK). 

For poling the composites, gold electrodes were deposited by sputtering on both sides of the cast films. Square samples of 5 mm side were then cut, and poling was carried out in a silicon oil bath under a field of 100 ± 10 kV/cm. Two procedures were used. In procedure P1 the poling field was applied for 30 min at 110 °C and during 1 h while cooling down to approximately 30 °C. In procedure P2, the electric field was switched off during the 1 h cooling to about 30 °C.

The d_33_ measurements were performed with an in-house-made Berlincourt-type apparatus. Each value was the average of three to four measurements. 

Capacitance and dielectric loss tangent were measured at room temperature between 10^2^ and 10^6^ Hz, with an applied voltage of 1 V_rms_ using an HP4194A impedance/gain-phase analyzer (Hewlett Packard, Palo Alto, CA, USA). Relative permittivity was calculated from capacitance knowing the electrode area and the thickness of the specimens. 

X-ray diffraction was performed on the films on a D8 DISCOVER diffractometer (Bruker AXS, Billerica, MA, USA) with CuKα radiation. The ratio of the intensities of the peaks corresponding to the (002) and (200) planes of barium titanate, appearing at 2θ angles of 44.9 and 45.3 degrees, respectively, was considered as indication of the polarization degree of the barium titanate particles.

Thermally stimulated discharge measurements were performed by heating poled composite specimens from 30 °C to 140 °C and then cooling down to 26 °C at a rate of 3 °C/min in a custom-made hot plate. The samples were placed on a Pt foil, insulated electrically from the hot plate with a sapphire wafer, and grounded. The current was collected from the top electrode of the sample and measured with a Model 486 picoammeter (Keithley Instruments Inc., Cleveland, OH, USA).

## 3. Results and Discussion 

### 3.1. Morphology

**Figure 2:**
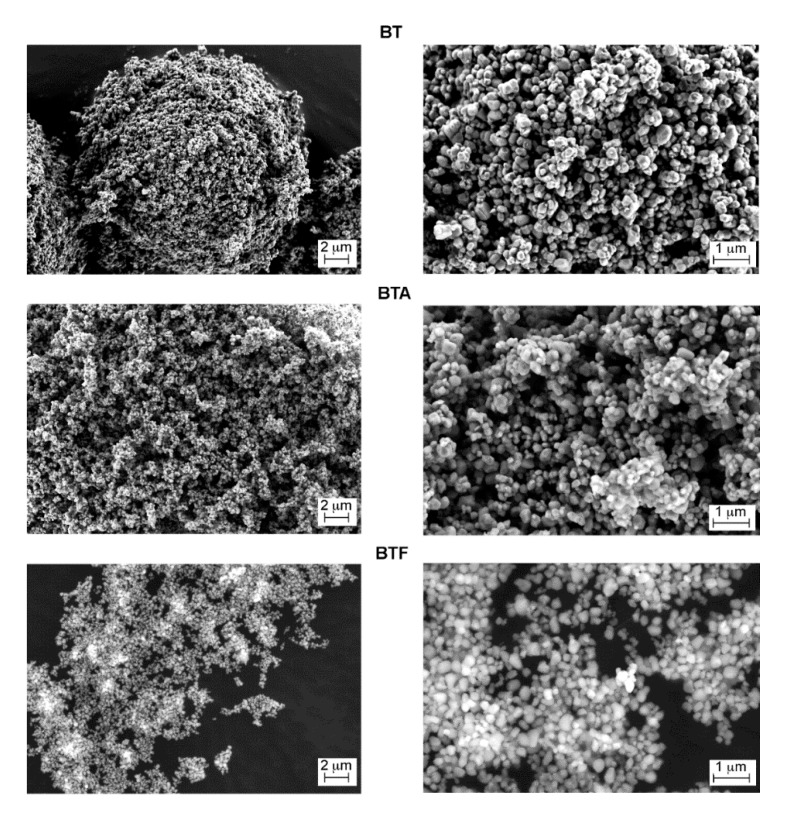
FESEM micrographs of BT, BTA, and BTF particles.

The barium titanate particles were observed in their pristine state and after surface modification with APTES or TFPTMS (Figure 2). A difference in the way particles were spreading on the SEM support can be noticed, the BT particles arranging in spherical macroagglomerates, while the surface-modified particles were more loosely spread on the carbon tape surface; this difference can be attributed to the reduced surface energy after silanization. However, at the microscale no relevant changes in the morphology could be observed, some micron-sized particle aggregates being visible in all cases. 

**Figure 3:**
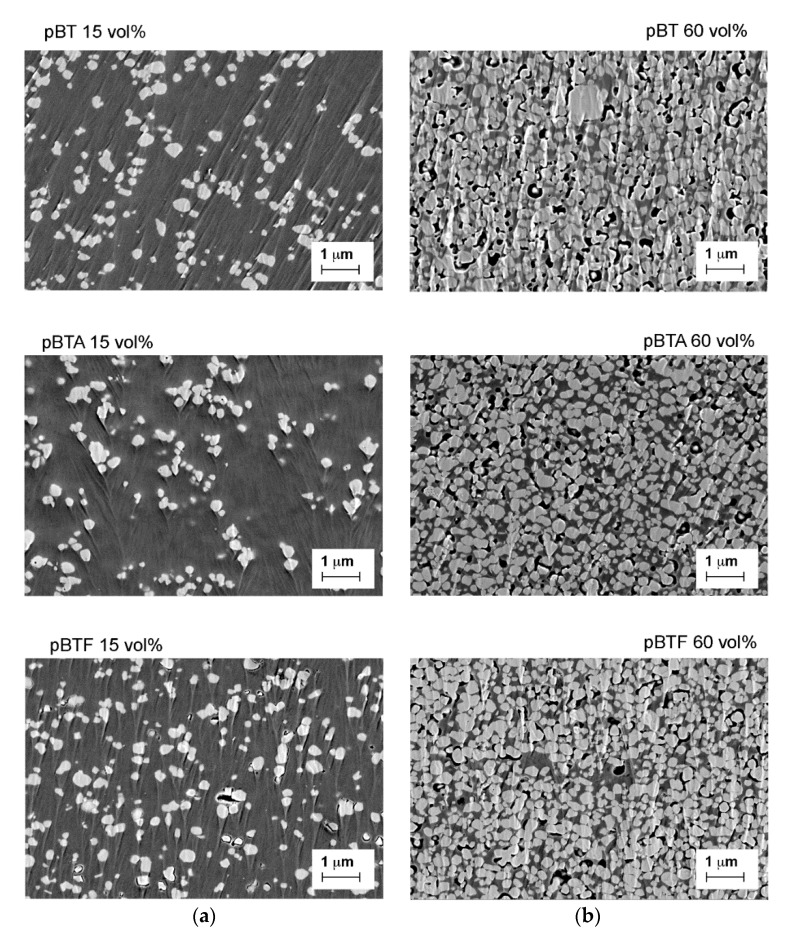
FESEM micrographs of cross-sections of composites with 15 vol.% (**a**) and 60 vol.% (**b**) of barium titanate particles, pristine or surface-modified.

The cross-sections of the composites with 15 vol.% and 60 vol.% of barium titanate particles, pristine or surface-modified, are shown in Figure 3. The particles were generally well-dispersed in the matrix, although some larger agglomerates, which were also associated with local porosity, could be identified in all cases (as better visible in Figure S1, showing lower magnification FESEM images). Among the composites with 15 vol.% of ceramics, pBTF showed more voids located at the particle-matrix interfaces than pBT and pBTA. The morphology of composites with 60 vol.% of ceramics was confirmed to be similar to all types of particles. Overall dispersion of the particles was good, with few larger aggregates visible in all cases. Some porosity was also present for all composites, both at the interface with the particles and within the matrix: porosity was found preferentially in correspondence to the larger aggregates, or at the bottom side of the films, presumably because the solvent could less easily escape during evaporation. By image analysis, the porosity was estimated to be 7.5, 6.4, and 4.5 vol.% for composites with 60 vol.% of BT, BTA, and BTF particles, respectively. In our previous work, we had found by density measurements for composites fabricated with the same procedure about 16 vol.% porosity with BT particles, 11 vol.% porosity with BTA particles, and 9 vol.% with BTF particles [21]. Despite a slight difference in the absolute value given by the two methods, in both cases, it was confirmed that the porosity was low considering the high volume fraction of filler, and that the porosity difference with respect to surface modification was negligible.

Furthermore, in our previous work we confirmed that the composites prepared with the procedure used here, independently from surface modification, for equal volume fractions of particles had similar crystallinity, checked by differential scanning calorimetry, and similar crystalline structures of the polymer and of the barium titanate, assessed by XRD [21].

### 3.2. Piezoelectric Response 

**Figure 4:**
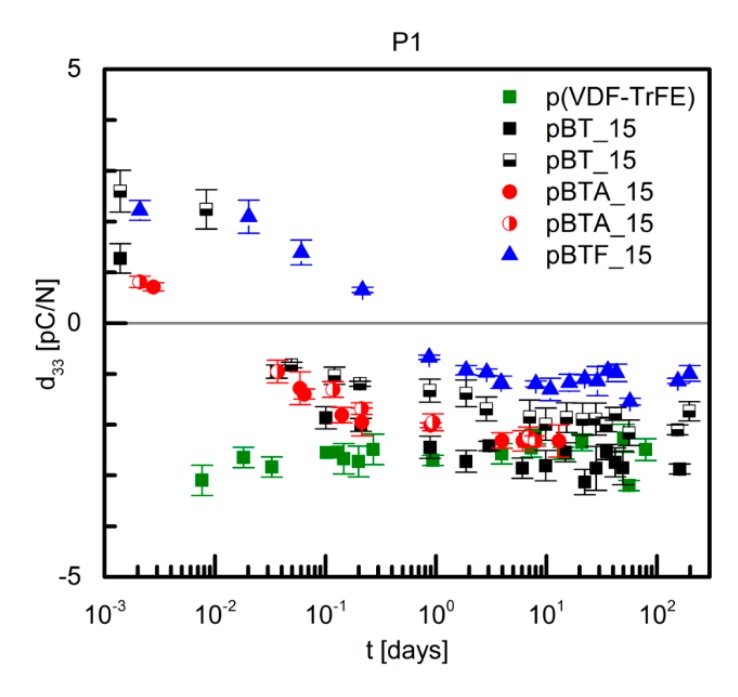
Piezoelectric coefficient d_33_ as a function of time of P(VDF–TrFE) and of P(VDF–TrFE)/BaTiO_3_ composites with 15 vol.% of ceramic particles, poled with procedure P1.

Figure 4 shows the time dependence of the measured d_33_ of P(VDF–TrFE) and composites with 15 vol.% of BaTiO_3_, and poled with procedure P1, i.e., applying the poling field of 100 kV/cm at 110 °C for 30 min and then for further 60 min while cooling to room temperature. For P(VDF–TrFE), the measured d_33_ was negative as expected, with a value around −3 to −2 pC/N, quite stable during the observed time of 100 days. As the poling field was much lower than the usually applied poling field for this polymer, this d_33_ value is very low with respect to the commonly reported d_33_ values [24,25]. Composite films with 15 vol.% of barium titanate particles, pristine and surface-modified, showed initially a modest positive d_33_, of about 1 to 3 pC/N, then within the first day the d_33_ value changed sign and reached a fairly stable value, of −1 to −3 pC/N, similar to that of the unfilled polymer (Figure 4). The negative sign of d_33_ indicates that the piezoelectric response was mainly due to the contribution of P(VDF–TrFE). The small differences in the absolute values of d_33_ between the different composites with 15 vol.% of ceramics are most likely attributable to small variations of the effective poling field applied. 

**Figure 5:**
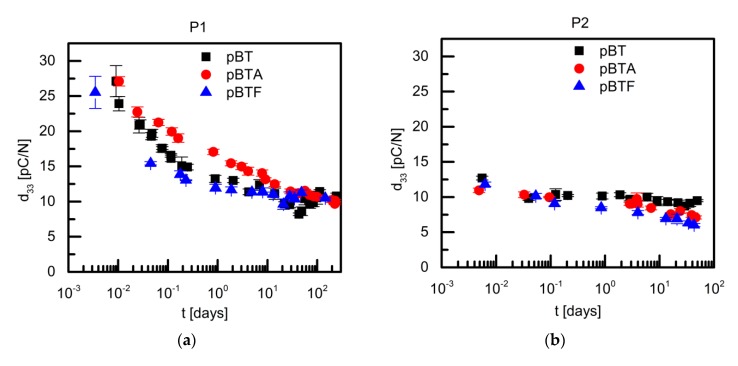
Piezoelectric coefficient d_33_ as a function of time of P(VDF–TrFE)/barium titanate composites with 60 vol.% of ceramics, poled with procedure P1 (**a**) and with procedure P2 (**b**).

Figure 5 shows the time dependence of d_33_ of P(VDF–TrFE) composites with 60 vol.% of BaTiO_3_, poled with procedures P1 and P2. In case of P1, the initial d_33_, measured within the first 5 min from poling, was between 25 and 28 pC/N; the measured d_33_ values then decreased in time, and after 100 days at room temperature, they had reached a value of about to 10 pC/N for all composites. However, the rate at which the d_33_ value decreased in time was quite different depending on the surface modification of the barium titanate particles. The composites containing BT and BTF particles behaved similarly: their d_33_ showed a very steep logarithmic decrease with time during the first two days, falling to about 50% of their initial value, and within one week they reached a plateau value equal to about 40% of their initial value. On the other hand, the d_33_ of pBTA composites dropped at a constant rate in the entire observed time interval: eventually, after more than 100 days at room temperature it had a value similar to that observed for pBT and pBTF composites, however, it did not seem to have reached a plateau value.

In a previous work, we compared solvent cast composites with 60 vol.% of BT and BTA particles, fabricated with a less efficient dispersing procedure, so that a large morphology improvement, and porosity decrease, had been obtained with surface-treated particles [23]. The poling procedure used was similar to P1 of this work. The initial d_33_ doubled when surface-modified particles were used instead of pristine particles, and in both cases, it decreased with time elapsed from poling. Similarly to what was shown in the present work, after one week the d_33_ of the composites containing pristine particles had reached a plateau value, while this was not the case for composites containing surface-treated particles, for which the decrease of d_33_ in time was slower. When the same composites were compression molded, hence porosity was negligible, the d_33_ values followed similar trends in time as for solvent cast composites, but the initial values were similar with pristine and modified particles, as it happened with the solvent cast composites studied in the present work. Hence, a comparison of our previous results with the ones obtained here suggests that the value of the initial d_33_ was mainly affected by porosity, while the evolution of d_33_ in time was influenced by the surface modification of the particles. 

In order to understand if the poling conditions would affect the temporal evolution of d_33_, the composites with 60 vol.% of ceramic particles were also poled with procedure P2, applying the poling field of 100 kV/cm at 110 °C during 30 min, and then cooling down to room temperature without field applied. In this case, for all composites, the initial d_33_ values of 10–13 pC/N were measured, which then decreased with moderate decay rates in the observed time range (Figure 5). In this case, due to the low initial d_33_ values, it was not possible to clearly understand if the decay rates changed in time, and further comments on this would be too speculative. It is anyhow clear that the application or not of the electric field during cooling to room temperature had a great impact on the measured d_33_ at short times from poling, and on its temporal evolution, although at longer times from poling the difference was less evident, at least for the field intensity used in this work. In what follows, we try to understand the origin of these differences by looking at the evolution of permittivity and of the poling degree of the barium titanate particles, and by thermally stimulated discharge measurements taken on poled samples.

### 3.3. Temporal Evolution of Relative Permittivity

The permittivity of P(VDF–TrFE) and composite films with 15 vol.% of ceramic particles, poled with procedure P1, as a function of time elapsed from poling, is shown in Figures S2 and S3 of the supporting information. Permittivity of the poled materials was higher than that of the corresponding unpoled materials. Increase of permittivity upon poling has been observed for PVDF, attributed to dipole alignment and preferred orientation in the interface regions between crystalline and non-crystalline regions [26]. On the other hand, after poling at room temperature with poling fields typically above 500 kV/cm, for annealed P(VDF–TrFE), permittivity was found to be lower than for the unpoled material, while no change was detected for not annealed P(VDF–TrFE) [27,28]. These findings were explained by attributing the difference of the dielectric constant in the poled and in the unpoled state mainly to a contribution of domain wall motion [28]. In our case, the permittivity of P(VDF–TrFE) was found to be higher after exposure to the poling procedure, which was carried out at conditions different from those typically used, namely at high temperature and with a much lower poling field. The permittivity of the poled P(VDF–TrFE) film decreased steadily with the logarithm of time, reflecting the disordering of the P(VDF–TrFE) dipoles. For the composites with 15 vol.% of ceramic particles, after the initial increase upon poling, the permittivity remained practically constant for a period of fewer than 0.1 days. Permittivity of barium titanate is larger in the unpoled state than in the poled state, because of the anisotropy of the dielectric tensor, with relative permittivity parallel to the c-axis much lower than parallel to the a-axes. Therefore, the decrease of permittivity due to depoling of P(VDF–TrFE) and its increase due to the depoling of barium titanate possibly compensated each other. Afterward, a logarithmic decrease with time was observed. Dispersion at low frequencies due to Maxwell–Wagner–Sillars (MWS) effect, i.e., to charge accumulation at the interfaces, was not noticeable for these materials, nor before poling, neither after poling.

**Figure 6:**
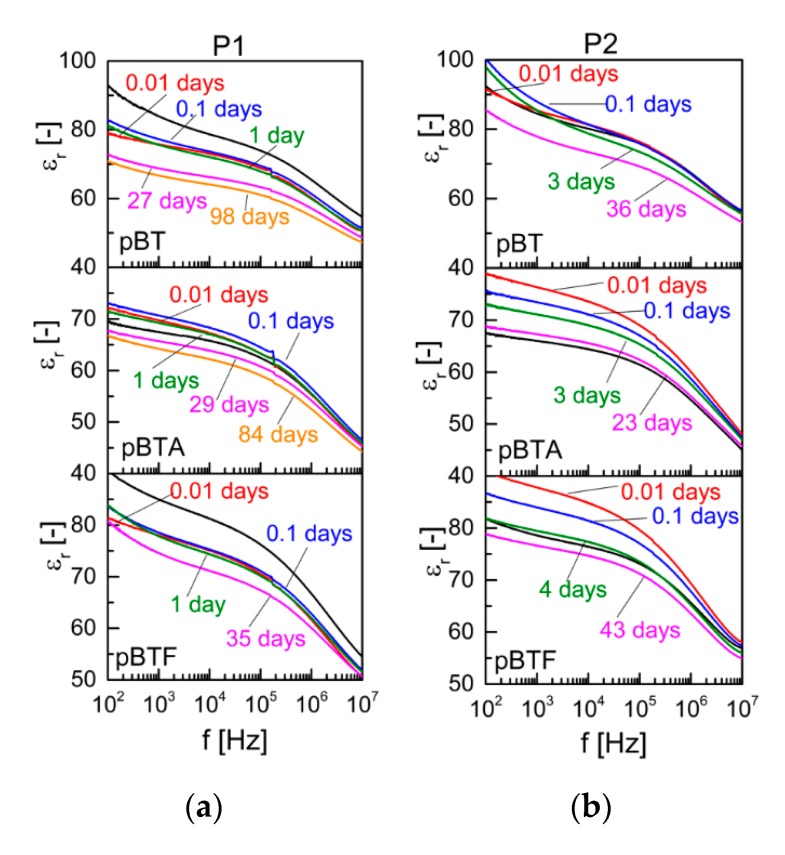
Permittivity composites with 60 vol.% of particles, measured at different times from poling, with poling procedures P1 (**a**) and P2 (**b**).

For the composites with 60 vol.% ceramics, some differences in the evolution of permittivity could be detected depending on the surface chemistry of the particles. Selected permittivity curves as a function of frequency, taken at different times from poling, are shown in Figure 6, and the evolution of permittivity in time, at selected frequencies, is represented in Figure S4 of the supporting information. For pBT, permittivity decreased upon poling, more markedly with P1 than with P2. For pBTA composites, instead, permittivity increased upon poling, very slightly with P1 and more evidently with P2. This difference may again reflect the competition between the permittivity changes of the polymeric and ceramic phases upon poling. For pBTF composites, comparison before and after poling was not possible as some small pieces detached during poling due to the fragility of the samples, leaving an irregular shape after poling which made it difficult to exactly calculate the area: permittivity after poling may, therefore, be somewhat underestimated. In the time elapsed after poling, for the three composites poled with P1, permittivity was stable or slightly increased for about one day, then showed a logarithmic decrease during the following time. The pBT and pBTF composites before poling showed dispersion at low frequency due to MWS effect; upon poling, the MWS effect was temporarily suppressed, but appeared again from 0.1 days onwards. The pBTA composites, on the other hand, did not show MWS dispersion before poling, and a slight MWS effect could be observed only after more than 80 days from poling. This suggests a different behavior for the charges accumulated at the interfaces, and redistribution of the charges appears also to happen differently when particles modified with aminosilane were used, versus those pristine or modified with fluorosilane. As the particles modified with the fluorosilane behaved similarly to pristine particles, and did not show the same effect as the ones modified with aminosilane, the different behavior may be ascribed to the different nature of the chemical group at the end of the silane chain: the amino group, in fact, may bear a positive charge, while this is not the case for the fluorine group. For the composites poled with P2, the permittivity decreased logarithmically with time, immediately for pBTA and pBTF, and as of 0.1 days for pBT. The MWS effect present for pBT and less markedly for pBTF also with P2 temporarily disappeared upon poling, and gradually reappeared again as soon as time elapsed.

The position of the resonance peak appearing on the relative permittivity curves of samples poled with P1 did not evolve with time after poling. Indeed, the resonance frequency *f* is linked to the density and stiffness of the material by the relationship $f\propto \left(1/h\right)\cdot \sqrt{\left(E/\rho \right)}$, where *h* is the linear dimension that determines the resonance frequency of the specimen, *ρ* is its density, and *E* is the effective elastic modulus [24]. In contrast, the height of the resonance peak (difference between permittivity at peak maximum and permittivity at peak minimum) decreased in time similarly to the d_33_, i.e. with a faster decay during the first day for the pBT composite, and a constant logarithmic decay in time for the pBTA composite. For the composites poled with P2, the resonance peak was barely visible as of the first measurements, due to the rather low d_33_ showed by those samples, and no further analysis was possible.

### 3.4. X-Ray Diffraction Analysis

**Figure 7:**
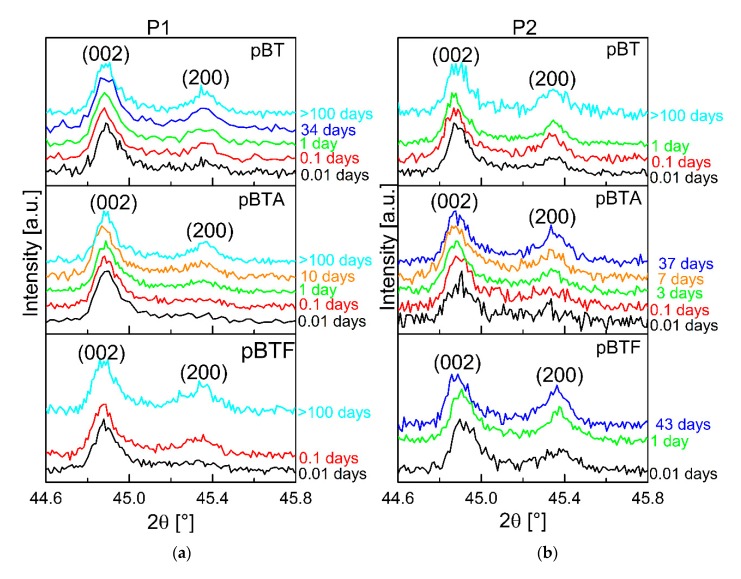
X-ray diffraction patterns at different times after poling for composites with 60 vol.% of particles, poled with P1 (**a**) and with P2 (**b**).

The XRD analysis gives insights regarding the poling degree of the BT particles inside the poled composites. For all composites, a decrease of the poling degree of barium titanate was observed. Diffractograms for the composites with 15 vol.% of ceramic particles are given in the supporting information (Figure S5). Selected diffractograms taken at different times from poling for composites with 60 vol.% of ceramic particles are shown in Figure 7. Firstly, the composites poled with P1 showed better poling of the barium titanate particles with respect to their homologs poled with P2. Poling consists of the reorientation of dipoles in P(VDF–TrFE) and of domains in BaTiO_3_. In a 0–3 composite, the poling field felt by BaTiO_3_ particles is screened by the polymer; therefore, it is smaller than the applied field. So, a long time is needed to orient as many domains as possible. In the P1 procedure, the electric field was applied also during cooling, so for a longer time than in the P2 procedure. If the poling field is removed at a high temperature, as done in P2, those domains that were oriented will tend to randomize because of the high thermal energy, and the sample will partially depole. Secondly, for both poling procedures, the barium titanate particles showed a marked depoling with time, indicated by the decrease of the I_002_/I_200_ ratio; the depoling rate was higher than expected based on the aging of poled barium titanate ceramics [22]. As a possible explanation, one can consider that aging of BaTiO_3_ is usually studied on samples with metallic electrodes. In these conditions, the polarization at the surface is completely screened: ideally, there is no depolarizing field, or it is relatively small. In the case of a 0–3 composite, this electrical boundary condition is completely different. The concentration of screening charges on the interface may be much smaller than for barium titanate with metallic electrodes, hence the depolarizing field inside particles may be larger, leading to faster depolarization. Finally, as it can be inferred by comparing the diffractograms taken at similar times for homologs, composites poled with the two procedures, the rise of the 200 peak, indicating depoling of BT, was faster for the composites poled with P2 than for those poled with P1. 

Comparing the diffractograms taken at 0.01 days for composites poled with P1, barium titanate appears to have retained more poling for pBTA than for pBT and pBTF. However, a clear correlation between the differences in the d_33_ decay rates depending on surface modification of the particles and the corresponding depoling rates could not be established, due to noise in the XRD data. Nevertheless, the XRD measurements in time clearly confirm that the depoling of the ceramic particles contributed to the decay of d_33_ in time.

### 3.5. Thermally Stimulated Discharge Current Analysis

**Figure 8:**
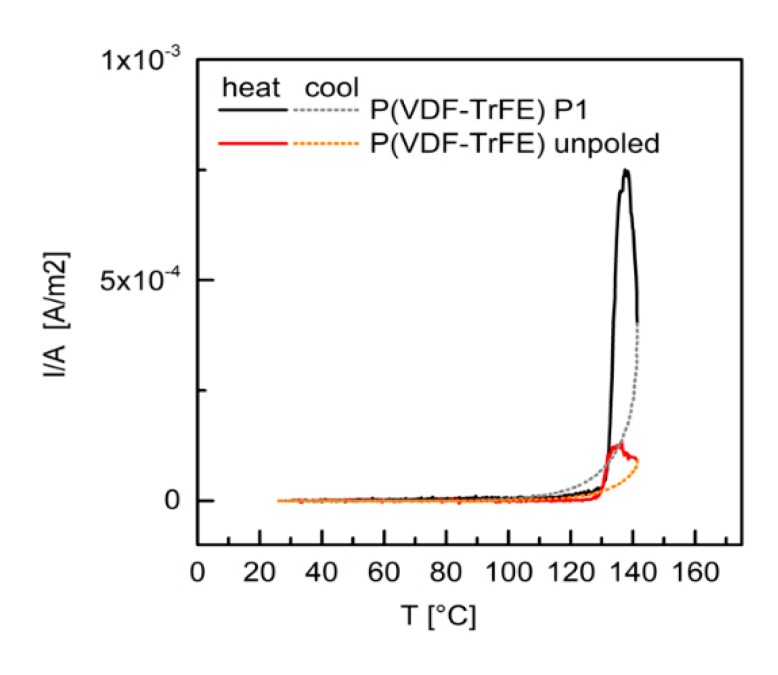
Thermally stimulated current discharge for P(VDF–TrFE) poled and unpoled. Full lines indicate the heating cycles and dotted lines the cooling cycles.

**Figure 9:**
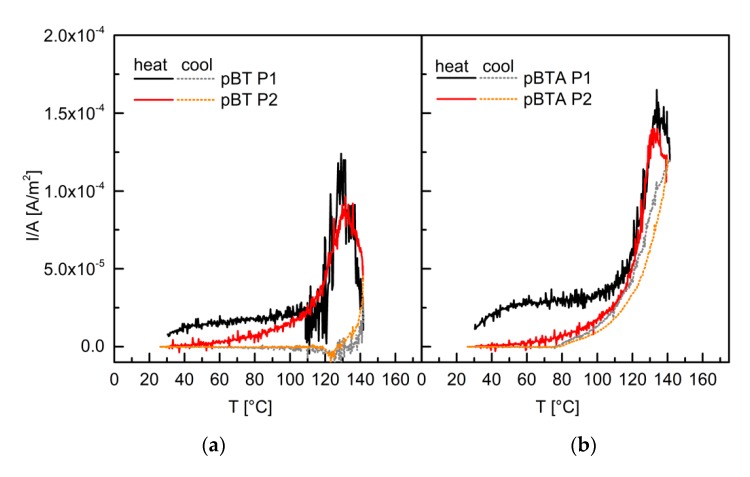
Thermally stimulated current discharge for pBT (**a**) and pBTA (**b**) composites with 60 vol.% of ceramics, poled with P1 and with P2. Full lines indicate the heating cycles and dotted lines the cooling cycles.

To better understand the role of charges in the mechanism underlying the different decay rate for d_33_ due to different modifications of the barium titanate particles, thermally stimulated discharge current analysis was carried out for the P(VDF–TrFE) not poled and poled with P1 (Figure 8), and for pBT and pBTA composites with 60 vol.% of ceramic particles (the pBTF composites were considered to behave similarly to the pBT ones), poled with P1 and P2 (Figure 9). When discussing these results, one should bear in mind that charges in these materials may have two origins: one from the polarization of barium titanate and of P(VDF–TrFE), and the other from trapped charges at interfaces. Depoling of barium titanate and P(VDF–TrFE) happens at their Curie temperatures (both around 130 °C), while trapped charges in general get released dominantly at the temperature of poling. In this case, the temperature of poling (110 °C) and the Curie temperatures are close, and these peaks may overlap.

For both the unpoled and the poled P(VDF–TrFE) films, a discharge current was measured with an onset above 120 °C. The current intensity was much larger for the poled polymer. This current discharge, which at maximum was in both cases close to 137 °C, corresponded to the depoling of P(VDF–TrFE) at the Curie temperature. Upon cooling, a positive current intensity was still detected, which decreased to zero around 90 °C. This means that some poling was retained also after heating above the Curie temperature. For the poled P(VDF–TrFE) only, a small current discharge (of the order of 10^-6^ A/m^2^) was observed during heating from room temperature to 120 °C and may be related to trapped charges, most likely at the polymer–electrode interfaces.

Both pBT and pBTA, composites poled with P1 showed current discharge during heating already from room temperature, and one order of magnitude larger than for the polymer alone. The current intensity was slightly larger for the composite containing BTA than for the one containing untreated particles. Then, above 110 °C, the discharge current intensity steeply increased, and a peak associated with the Curie transitions of barium titanate and P(VDF–TrFE) appeared for both composites, around 130–135 °C; for the pBTA composites, the maximum of the current discharge peak was higher than for the pBT composite; for both composites, however, the maximum current intensity was much lower than for the poled P(VDF–TrFE). During the cooling cycle, the behavior of the composites was different depending on the type of particles. For the pBT composites, the current intensity decreased and then changed sign around 135 °C, passed through a slightly negative current intensity minimum (between 130 °C and 125 °C), and finally vanished for temperatures below 100 °C. On the other hand, for the pBTA composite, the current intensity during cooling was always positive and did not decrease to zero until below 80 °C. This indicates that the pBT composites depoled effectively as soon as the Curie temperature was reached, while the pBTA composites retained some poling and charges even after reaching the Curie temperature. The behavior of the pBTA composites was in this respect more similar to that of the unfilled polymer.

For the composites, both with untreated and aminosilane-treated particles, poled with P2, the current discharge in the heating cycles for temperatures lower than 110 °C, seen for the composites poled with P1, did not appear. Only the tail of the main discharge peak was visible below 110 °C. The temperature at the peak maximum was close to 132 °C for both the pBT and pBTA composites, and the maximum intensity was slightly lower than for the corresponding composites poled with P1. The behavior during the remaining part of the heating cycle, and during the cooling cycle, looked then similar to what was described for the corresponding composites poled with P1.

The difference in current discharge below 110 °C between the two poling procedures suggests that charges were trapped with procedure P1, due to the cooling under the electrical field, which were then partly released already at low temperatures. These charges may create an electret effect, at the polymer–particles interface and/or at the polymer–electrode interface, which would not be present for composites poled with P2. 

Furthermore, the current density was higher for the pBTA composites than for pBT composites, for both poling procedures, indicating that more charges were trapped with aminosilane-treated particles than with pristine particles; the delay in zeroing the current during the cooling cycle indicates also for pBTA a higher thermal stability of the trapped charges, either coming from polarization or trapped at interfaces, which were retained also at temperatures higher than the Curie temperature. This different thermal stability of the charges due to the different chemical groups present at the surface of the ceramic particles may have an effect on the evolution of the piezoelectric coefficient in time. 

## 4. Conclusions

In this study, we compared composites with similar morphologies and crystalline structures prepared with barium titanate particles having different surface chemistries, and following two different poling procedures. Attention was paid to the long-term evolution of the piezoelectric coefficient d_33_, permittivity, and poling state of barium titanate particles. For the first time, we highlighted that depending on the nature of the coupling agent used, effects due to different charge trapping may be induced, which also depends on the poling procedure used. These effects may alter the decay rate of the measured d_33_ and may increase up to above 100 days, the time needed to reach a stable value. This study is a first attempt to elucidate the underlying mechanism, and highlights the need to take into account the effect of the surface chemistry of inorganic particles when selecting coupling agents to compatibilize these particles with polymer matrices.

## Supplementary Materials

The following are available online at https://www.mdpi.com/1996-1944/13/1/225/s1, Figure S1: FESEM micrographs of cross-sections of composites with 15 vol.% and 60 vol.% of barium titanate particles, pristine or surface-modified, Figure S2: Evolution of permittivity in time, at selected frequencies, of P(VDF–TrFE) before poling (hollow symbols) and poled with procedure P1 (full symbols). Logarithmic trendlines are also reported as a guide for the eyes. Figure S3: Evolution of permittivity in time, at selected frequencies, of composites with 15 vol.% of ceramic particles before poling (hollow symbols) and poled with procedure P1 (full symbols). Logarithmic trendlines are also reported as a guide for the eyes. Figure S4: Evolution of permittivity in time, at selected frequencies, of composites with 60 vol.% of ceramic particles before poling (hollow symbols) and poled with procedure P1 (full symbols). Logarithmic trendlines are also reported as a guide for the eyes. Figure S5: X-ray diffractograms for the pBT and pBTF composites with 15 vol.% of ceramic particles. Time from poling in days is given in the legend.
