# Supplementary material for: Interface-Dominated Time-Dependent Behavior of Poled Poly(Vinylidene Fluoride–Trifluoroethylene)/Barium Titanate Composites
Source: Materials (Basel). 2020 Jan 4;13(1):225. doi: 10.3390/ma13010225 (PMC6981695; doi:10.3390/ma13010225)
Supplement: Supplementary file 1 [file materials-13-00225-s001.pdf]

materials

Supplementary Materials

Interface-Dominated Time-Dependent Behavior of Poled Poly(Vinylidene Fluoride–Trifluoroethylene)/Barium Titanate Composites

Sara Dalle Vacche [1,*,†], Dragan Damjanovic [2], Véronique Michaud [1] and Yves Leterrier [1,*]

[1] Laboratory for Processing of Advanced Composites (LPAC), Ecole Polytechnique Fédérale de Lausanne, EPFL-STI-IMX-LPAC, Station 12, CH-1015 Lausanne, Switzerland; veronique.michaud@epfl.ch

[2] Group for Ferroelectrics and Functional Oxides, Ecole Polytechnique Fédérale de Lausanne, EPFL-SCI-STI-DD, Station 12, CH-1015 Lausanne, Switzerland; dragan.damjanovic@epfl.ch

* Correspondence: sara.dallevacche@polito.it (S.D.V.), yves.leterrier@epfl.ch (Y.L.)

† Current address: Department of Applied Science and Technology, Politecnico di Torino, C.so Duca degli Abruzzi 24, 10132 Turin, Italy

Received: 30 November 2019; Accepted: 1 January 2020; Published: date

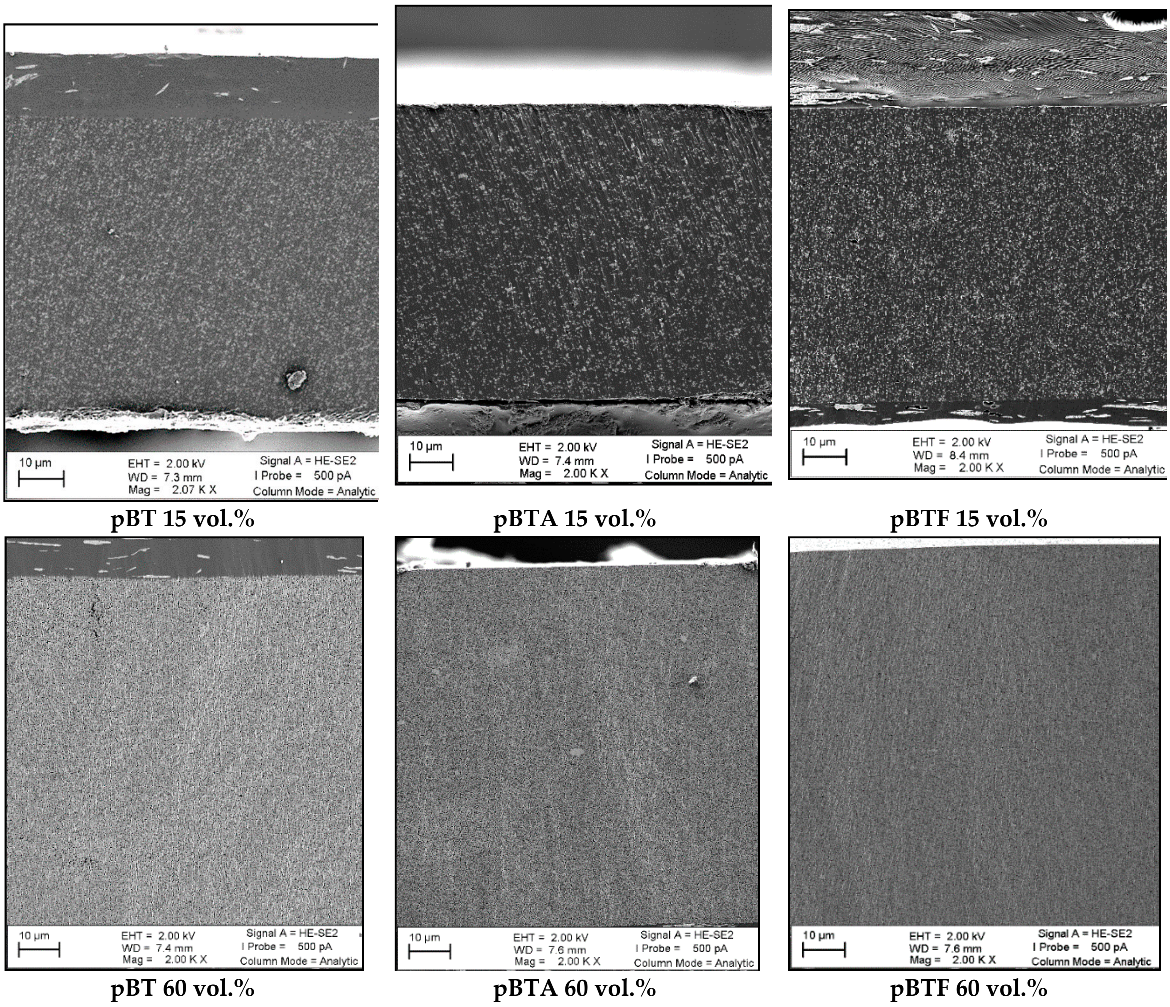

Figure S1. FESEM micrographs of cross-sections of composites with 15 vol.% and 60 vol.% of barium titanate particles, pristine or surface modified.

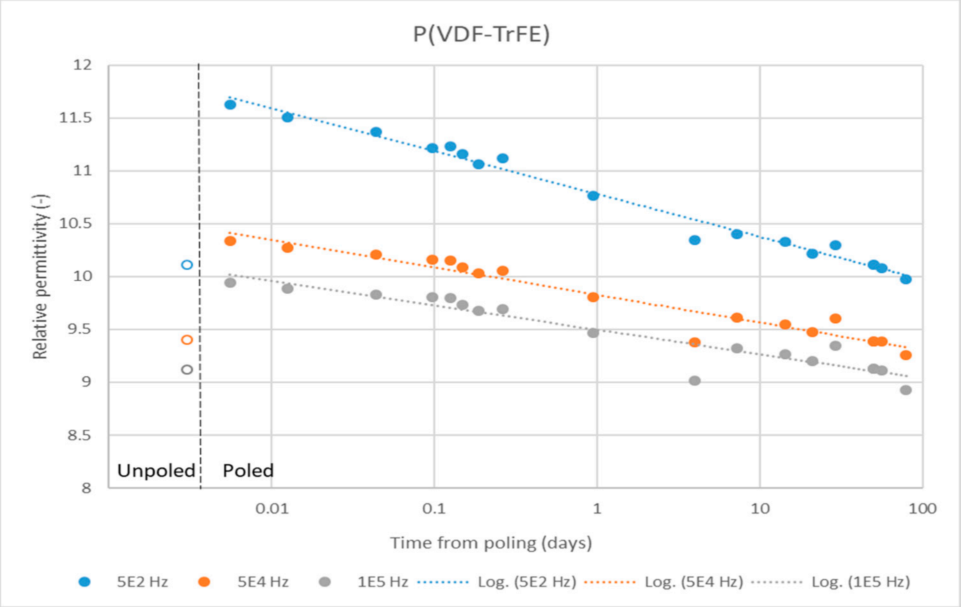

Figure S2. Evolution of permittivity in time, at selected frequencies, of P(VDF-TrFE) before poling (hollow sysmbols) and poled with procedure P1 (full symbols). Logarithmic trendlines are also reported as a guide for the eyes.

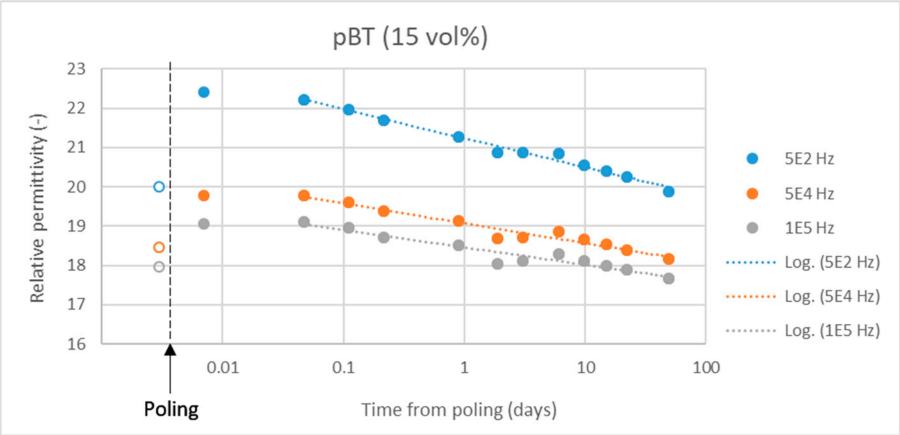

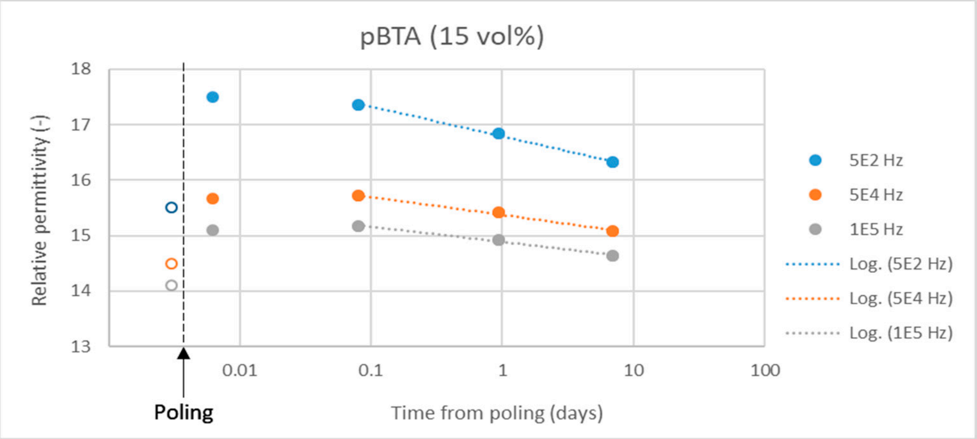

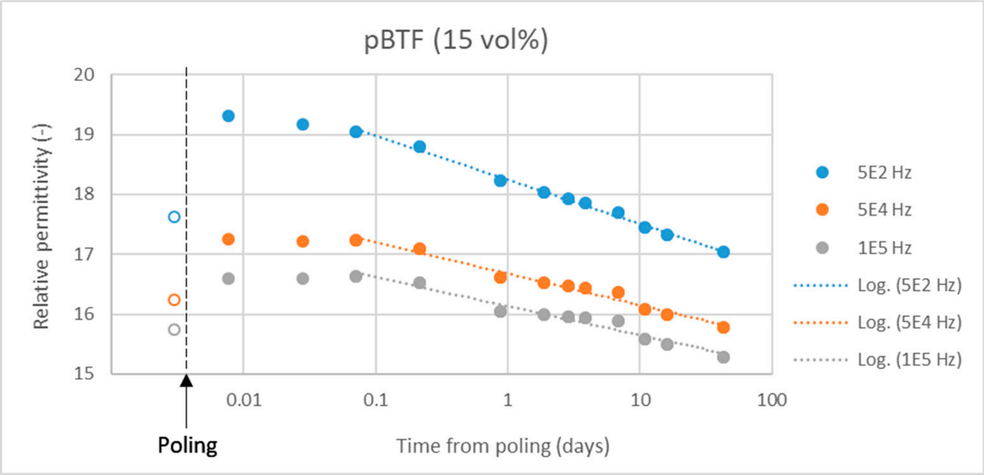

Figure S3. Evolution of permittivity in time, at selected frequencies, of composites with 15 vol.% of ceramic particles before poling (hollow sysmbols) and poled with procedure P1 (full symbols). Logarithmic trendlines are also reported as a guide for the eyes.

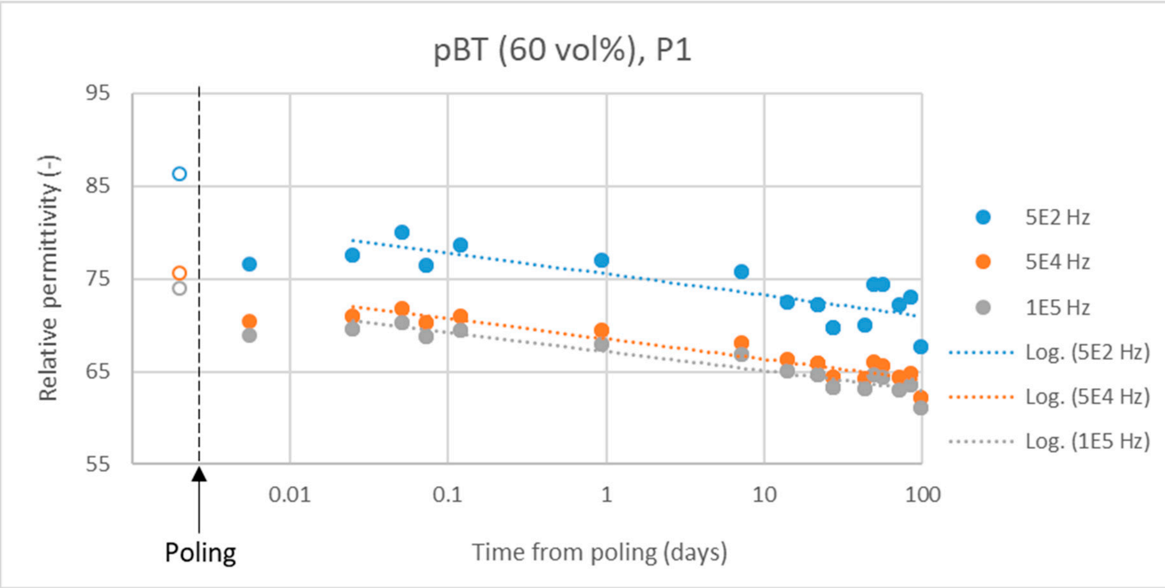

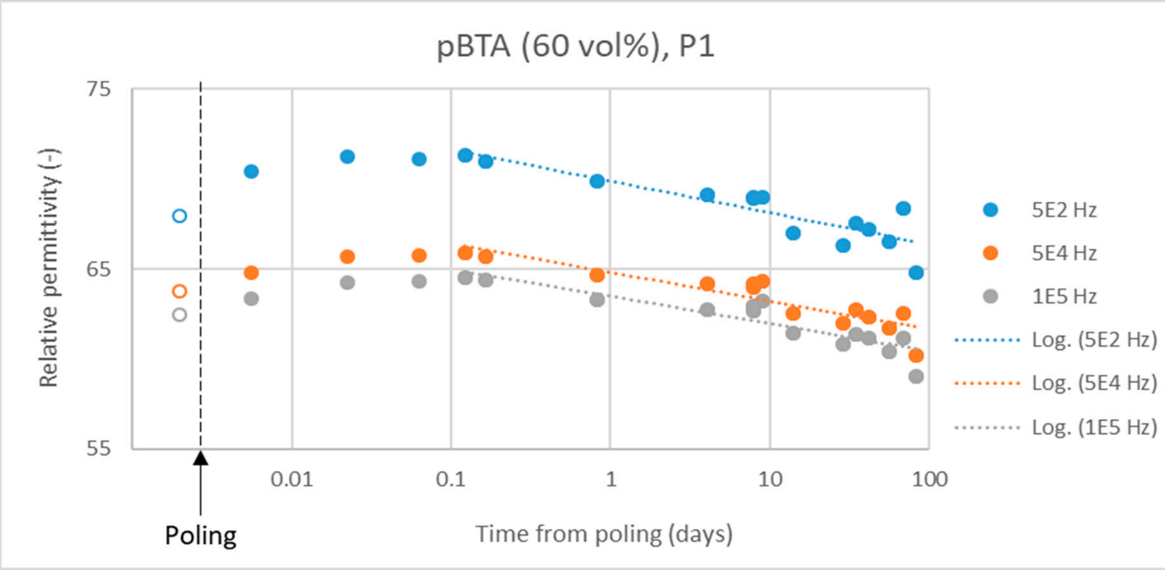

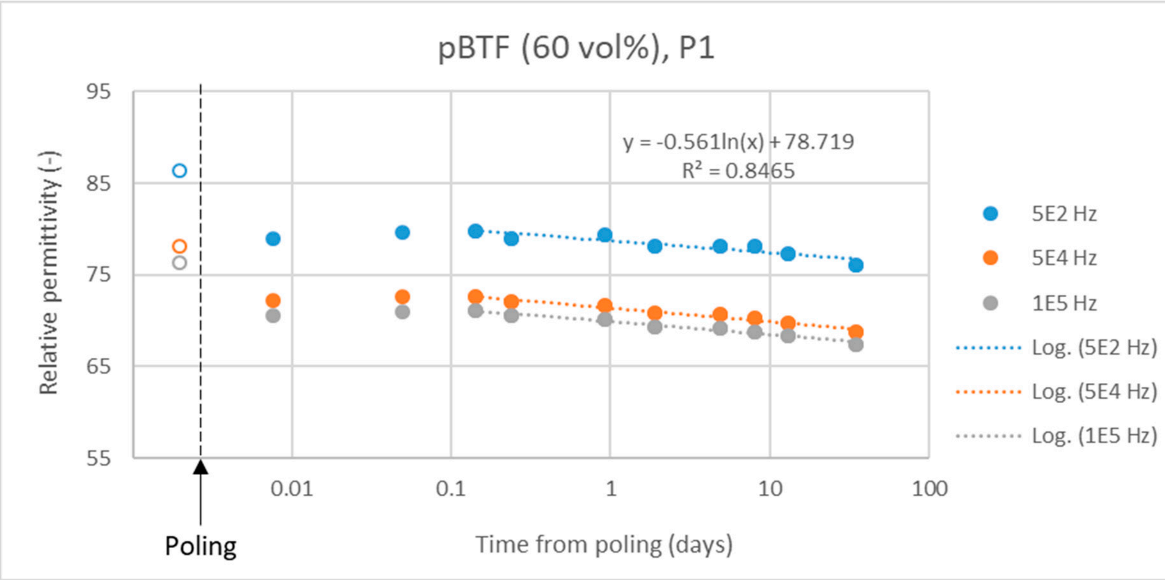

Figure S4. Evolution of permittivity in time, at selected frequencies, of composites with 60 vol.% of ceramic particles before poling (hollow sysmbols) and poled with procedure P1 (full symbols). Logarithmic trendlines are also reported as a guide for the eyes.

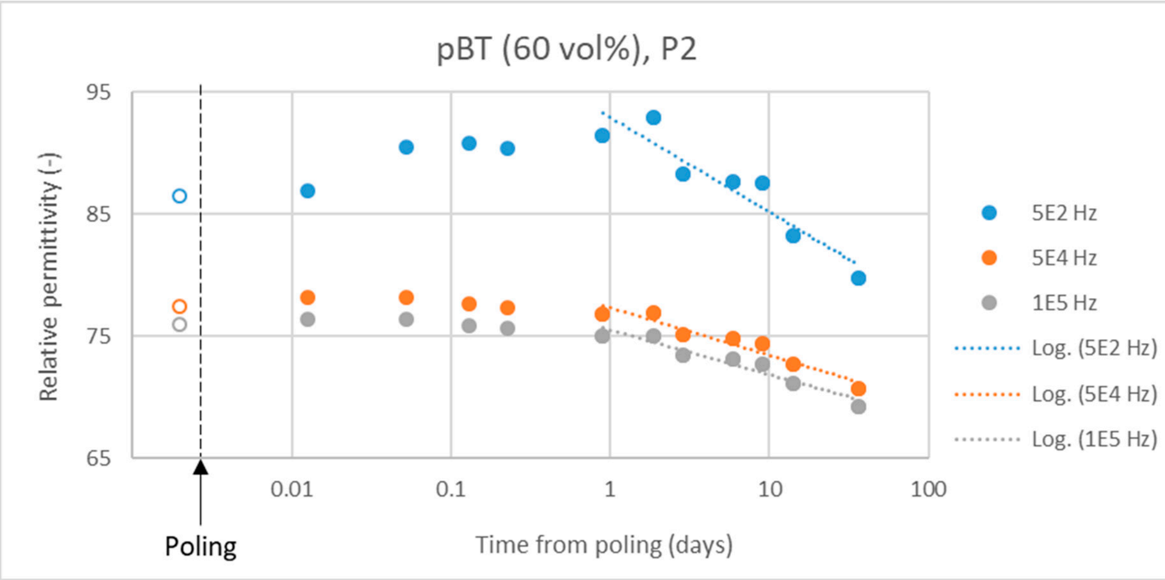

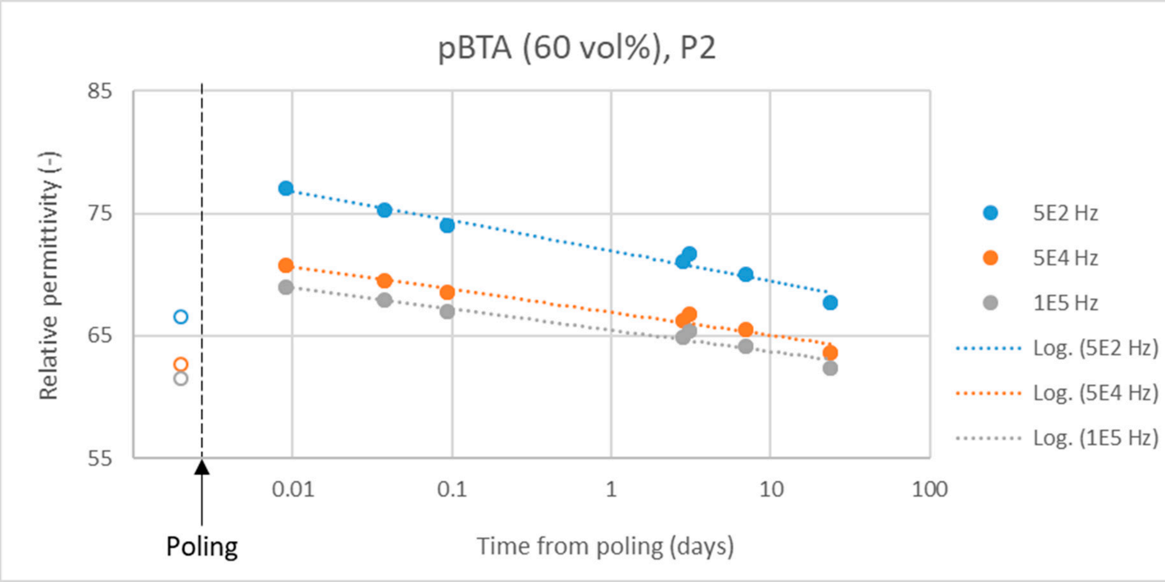

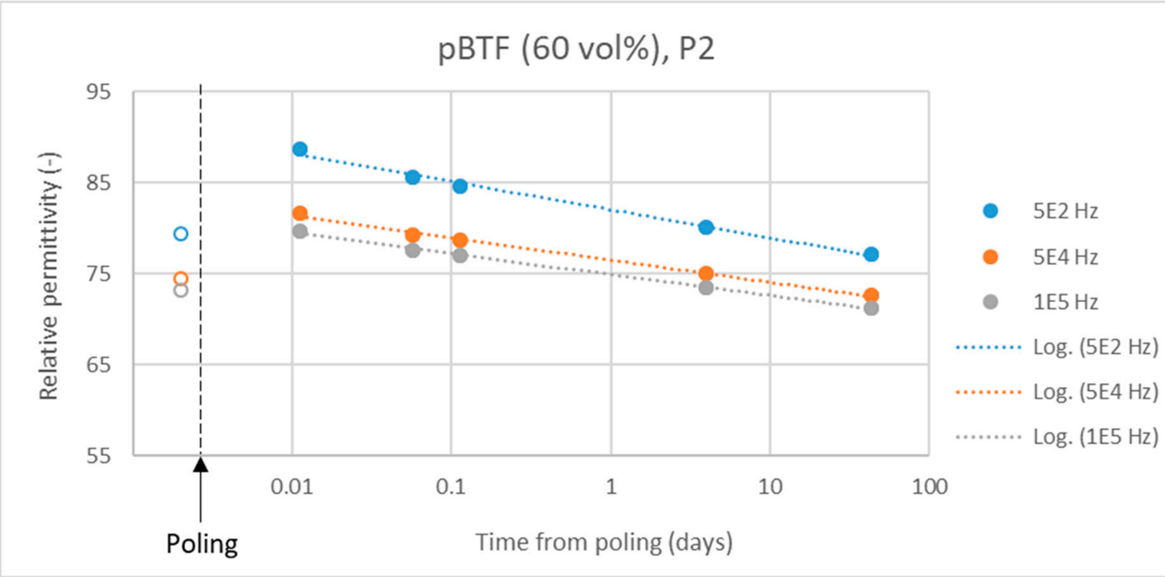

Figure S5. Evolution of permittivity in time, at selected frequencies, of composites with 60 vol.% of ceramic particles before poling (hollow sysmbols) and poled with procedure P1 (full symbols). Logarithmic trendlines are also reported as a guide for the eyes.

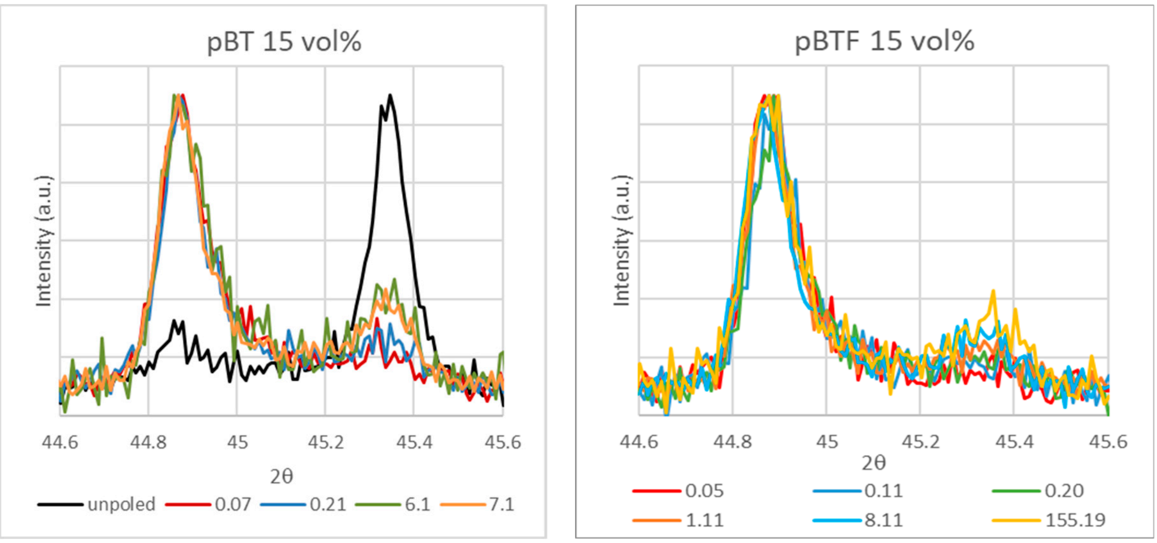

Figure S6. X-ray diffractograms for the pBT and pBTF composites with 15 vol.% of ceramic particles. Time from poling in days is given in the legend.

© 2020 by the authors. Submitted for possible open access publication under the terms and conditions of the Creative Commons Attribution (CC BY) license (http://creativecommons.org/licenses/by/4.0/).
